# Effects of *Bacillus amyloliquefaciens*- and *Bacillus licheniformis*-Fermented Feeds on Performance, Meat Quality and Gut Microbiot of Fattening Pigs

**DOI:** 10.3390/ani16142179

**Published:** 2026-07-13

**Authors:** Weizhi Wu, Yongben Wang, Youli Yao, Kejiang Jia, Xuan Luo, Lei Wang, Guofang Wu

**Affiliations:** 1 College of Animal Husbandry and Veterinary Sciences, Qinghai University, Xining 810000, China; 18534278919@163.com (W.W.); 13613828741@163.com (Y.W.); jiakejiang123@163.com (K.J.); cauluoxuan@163.com (X.L.); wanglei19831002@gmail.com (L.W.); 2 Qinghai Provincial Plateau Livestock Genetic Resources Protection and Innovative Utilization Laboratory, Xining 810000, China; 15709833353@163.com; 3 Qinghai-Tibet Plateau Key Laboratory of Animal Genetics and Breeding, Ministry of Agriculture and Rural Affairs, Xining 810000, China

**Keywords:** *Bacillus*, fermented feed, DLY crossbred pigs, growth performance, 16S rRNA, microbial community

## Abstract

This study aimed to evaluate the nutritional composition and fermentation quality of feed fermented by *Bacillus amyloliquefaciens* and *Bacillus licheniformis* as well as their effects on growth performance, meat quality, blood parameters, and gut microbiota in fattening pigs. A total of 72 healthy Duroc × Landrace × Large White crossbred barrows (68.78 ± 1.41 kg, *p* > 0.05 for initial body weight among groups) were randomly assigned to three dietary treatments, with four replicates pens per treatment and six pigs per pen. The pigs were fed one of three diets: (1) CK: 100% basal diet; (2) YR1: 50% basal diet + 50% feed fermented with *Bacillus amyloliquefaciens* YR3.2; and (3) YR2: 50% basal diet + 50% feed fermented with *Bacillus licheniformis* YR3.1. The experiment consisted of a 7-day adaptation period and a 50-day treatment period. The results showed that the crude protein, starch, lactic acid, acetic acid, and propionic acid contents were significantly higher in the treatment groups than in the control group (*p* < 0.05). The final body weight of fattening pigs, serum superoxide dismutase activity, and redness value of the *longissimus dorsi muscle* were significantly higher in the treatment groups than in the control group (*p* < 0.05). The carcass weight of the YR1 group was significantly higher than that of the other groups (*p* < 0.05). Moreover, the shear force of pork was significantly lower in the treatment groups than in the control group (*p* < 0.05). Microbial analysis showed that *Firmicutes* dominated the jejunum and cecum in all groups (>80%). In the YR1 group, the abundance of *Clostridium* increased in the jejunum, whereas the abundance of *Terrisporobacter* decreased. In the YR2 group, the abundances of both *Clostridium* and *Terrisporobacter* increased in the jejunum and cecum. In conclusion, fermented feed prepared with *Bacillus amyloliquefaciens* and *Bacillus licheniformis* has a positive effect on the growth performance of fattening pigs, but fermented feed prepared by *Bacillus amyloliquefaciens* YR3.2 has a more significant effect on improving growth performance, feed conversion efficiency and some meat quality indicators.

## Simple Summary

Selected *Bacillus* strains, such as *Bacillus amyloliquefaciens* and *Bacillus licheniformis*, have been used in feed fermentation because of their potential to improve feed nutritional quality and produce fermentation metabolites that may support intestinal health. In this study, 50% *Bacillus amyloliquefaciens* YR3.2 fermented feed and 50% *Bacillus licheniformis* YR3.1 fermented feed were added to the diet of ternary hybrid pigs in the fattening stage to replace the basic feed. Compared with the 100% basal diet control group, adding 50% *Bacillus amyloliquefaciens* YR3.2 fermented feed and 50% *Bacillus licheniformis* YR3.1 fermented feed improved growth performance, slaughter index, meat tenderness, antioxidant capacity and some immune indexes. The addition of fermented feed also changed the relative abundance of intestinal *Firmicutes*, *Bacteroidetes*, *Terrisporobacter*, *Faecousia* and *Clostridium_T* and regulated intestinal flora to promote body health. At present, there are few studies on the application of *Bacillus amyloliquefaciens* YR3.2 fermented feed and *Bacillus licheniformis* YR3.1 fermented feed in the fattening stage of ternary hybrid pigs. The results of this study provide a reliable and practical reference for *Bacillus* fermented feed instead of traditional feed to feed ternary hybrid pigs in fattening period and lay a solid scientific foundation for green and healthy breeding.

## 1. Introduction

Animal husbandry plays an important role in global food production, and its production efficiency is closely associated with animal health, feed utilization, and disease resistance [1,2]. In pig production, the fattening stage is directly related to market weight, feed cost, carcass yield, and final economic return. Therefore, nutritional strategies that improve growth performance, feed efficiency, and health status during this period are of practical importance for pig farming [3,4].

With the gradual ban of antibiotic growth promoters in feed in many countries and regions worldwide, the livestock industry is facing unprecedented challenges in disease prevention and productivity maintenance [5]. In this context, selected probiotic strains have received increasing attention as potential alternatives to antibiotics. Among them, spore-forming *Bacillus* strains are widely used not only in pigs but also in poultry, ruminants, and aquaculture animals because of their strong environmental tolerance, storage stability, and ability to produce digestive enzymes and functional metabolites [6,7]. However, the beneficial effects of *Bacillus* are strain-specific and may vary depending on animal species, diet composition, physiological stage, and feeding conditions [8]. Therefore, the effects of specific *Bacillus* strains need to be evaluated in the target animal species before practical application.

Selected probiotic *Bacillus* strains are spore-forming microorganisms with strong environmental adaptability and can survive under adverse conditions such as high temperature and acidity, making them suitable for feed fermentation processes [9]. During fermentation, these strains can produce extracellular enzymes and functional metabolites, thereby improving feed digestibility, enhancing nutrient bioavailability, and regulating intestinal microecological balance [10]. In addition, probiotic *Bacillus* strains may exert beneficial effects by inhibiting harmful bacteria, strengthening intestinal barrier function, and modulating host immune responses, which contribute to improved growth performance and disease resistance in animals [11]. Among these probiotic candidates, *Bacillus amyloliquefaciens* YR3.2 and *Bacillus licheniformis* YR3.1 have potential advantages for improving the nutritional quality of fermented feed. *Bacillus amyloliquefaciens* YR3.2 showed potential advantages in improving crude protein availability and reducing acid detergent fiber and NH_3_-N levels during fermentation, suggesting a stronger capacity for protein and fiber degradation [12,13]. *Bacillus licheniformis* YR3.1 showed a greater advantage in starch accumulation, indicating its potential role in improving the available energy fraction of fermented feed. These strain-specific fermentation characteristics suggest that the two *Bacillus* strains may improve feed quality through different pathways [14]. However, their effects on fattening pigs, especially in terms of growth performance, meat quality, and intestinal microbial composition, remain to be further clarified.

In this study, *Bacillus amyloliquefaciens* and *Bacillus licheniformis* were used as fermentation strains to produce bio-fermented feed by thoroughly fermenting a complete diet. This experiment was conducted to study the effects of diets containing the fermented feed on growth performance, carcass traits, meat quality, serum biochemical indexes, serum antioxidant capacity and serum immune function of fattening pigs [15]. In addition, 16S rRNA gene high-throughput sequencing was performed to systematically analyze the effects of dietary *Bacillus*-fermented feed on the microbial communities in the jejunal and cecal contents of fattening pigs.

## 2. Materials and Methods

### 2.1. Strain Source

*Bacillus amyloliquefaciens* YR3.2 and *Bacillus licheniformis* YR3.1 used in this experiment were high-quality strains isolated, screened, and cultured by the Plateau Livestock Protection and Utilization Laboratory of the Qinghai Academy of Animal Science and Veterinary Medicine. The strains were maintained as glycerol stocks at −80 °C before activation. The strains have not been deposited in a public culture collection.

### 2.2. Activation of the Bacteria

The glycerol stocks of *Bacillus amyloliquefaciens* YR3.2 and *Bacillus licheniformis* YR3.1 stored at −80 °C were thawed and streaked onto Luria–Bertani (LB) agar plates. The plates were incubated at 37 °C for 24 h. A single well-isolated colony of each strain was then picked and inoculated into LB broth, followed by incubation at 37 °C with shaking at 180 rpm for 12–16 h to obtain the activated bacterial culture. Subsequently, 100 μL of each activated bacterial suspension was inoculated into 1 L of LB broth for seed culture preparation. The inoculated cultures were incubated at 37 °C with shaking at 180 rpm for 36 h. When the viable bacterial count reached at least 1 × 10^7^ CFU/mL, the culture was used as a qualified *Bacillus* seed suspension for feed fermentation. The Luria–Bertani broth and Luria–Bertani agar plates (all raw materials were purchased from Yangling Zhongmei Biological Equipment Distribution Department, Xianyang, China) are prepared as follows:

(1) LB broth (g/L): yeast extract 5.0 g, peptone 10.0 g, sodium chloride 10.0 g, pH 7.0.
(2) LB agar plates (g/L): yeast extract 5.0 g, peptone 10.0 g, sodium chloride 10.0 g, pH 7.0, agar 15.0 g.

### 2.3. Feed Preparation

The complete diet was formulated using corn, soybean meal, wheat bran, and a premix. The premix was purchased from Jiangxi Cargill Feed (Yichun) Co., Ltd. (Yichun, Jiangxi, China), and the remaining feed ingredients were provided by Qinghai Yufu Animal Husbandry Co., Ltd. (Xining, Qinghai, China). The composition and nutrient levels of the basal diet (on a dry matter basis) are presented in Table 1.

The two bacterial seed suspensions were used separately to prepare fermented feeds, and no mixed-strain fermentation was performed. For each fermented feed, complete feed and water were mixed at a mass ratio of 1:1 to adjust the moisture content. Then, 5% (*v*/*w*) bacterial seed suspension was added based on the weight of the complete feed and thoroughly mixed to ensure uniform distribution of the strain in the feed substrate. The mixture was packed into fermentation bags equipped with one-way exhaust valves, sealed (Fermentation bags were purchased from Yangling Zhongmei Biological Equipment Distribution Department, Xianyang, China.), and fermented at room temperature (25 ± 2 °C) for 10 d. During fermentation, the bags were kept in a clean and shaded environment and were not opened to reduce the risk of contamination. The CK group consisted of feed mixed with water at a ratio of 1:1 without bacterial inoculation. The YR1 group was prepared using feed fermented with *Bacillus amyloliquefaciens* YR3.2, whereas the YR2 group was prepared using feed fermented with *Bacillus licheniformis* YR3.1. After fermentation, three independent replicate fermentation bags were prepared for each treatment, with each bag regarded as an independent replicate sample. Approximately 200 g of feed was collected from multiple locations within each bag, thoroughly mixed, transferred into sterile sealed sampling bags, and labeled for subsequent analysis of nutrient composition and fermentation-quality-related parameters.

### 2.4. Treatment Animals, Husbandry Management, and Treatment Design

This experiment was carried out in Qinghai Yufu Agricultural and Animal Husbandry Science and Technology Development Co., Ltd., Tianjiazhai Town, Huangzhong District, Xining City, Qinghai Province, from March 2025 to May 2025. The total experimental period was 57 days. A total of 72 healthy Duroc × Landrace × Large White crossbred barrows with an initial body weight of 68.78 ± 1.41 kg were used in this study. All pigs were ranked according to their initial body weight and then randomly allocated to three dietary treatments, with four replicate pens per treatment and six pigs per pen. Each pen was considered one experimental replicate. The control group (CK) was fed with 100% basic feed, the YR1 group was fed with 50% basic feed + 50% *Bacillus amyloliquefaciens* YR3.2 fermented feed, and the YR2 group was fed with 50% basic feed + 50% *Bacillus licheniformis* YR3.1 fermented feed. The feed ratios for all groups were based on dry matter. The experiment included a 7-day pre-feeding period and a 50-day trial period.

The treatment facility was provided by Qinghai Yufu Animal Husbandry Co., Ltd. Before the experiment, all pigs were clinically examined by farm veterinarians. Only pigs with normal appetite, normal behavior, and no obvious clinical signs of disease were included in the study. Vaccination records were checked before grouping to ensure that all pigs had completed the routine immunization program. According to the farm vaccination records, the pigs had been vaccinated against porcine reproductive and respiratory syndrome, foot-and-mouth disease, classical swine fever, and Mycoplasma hyopneumoniae pneumonia. The pigs were housed in a conventional mechanically ventilated pig house. Each pen provided at least 1.5 m^2^ of floor space per pig, and the barn temperature was maintained at approximately 20–25 °C during the experiment. Before the trial, the pig house, pens, feeders, drinkers, and related facilities were thoroughly cleaned and disinfected. During the experiment, all pigs were managed under the same housing and environmental conditions. Feed was provided using a pen-based trough feeding system. Pigs had free access to feed and were fed twice daily, at 07:30–08:30 and 18:30–19:30. Feed offered and feed refusals were recorded daily on a pen basis. Fresh drinking water was supplied ad libitum through automatic nipple drinkers throughout the experiment. During the experiment, the health status of pigs was monitored daily by farm staff and veterinarians. Appetite, behavior, fecal condition, respiratory signs, skin condition, lameness, and other abnormal clinical signs were observed and recorded. The feeding management, health monitoring, and vaccination procedures were consistent among all treatment groups.

At the end of the experiment, three barrows with body weights close to the average weight of each pen were selected for each replicate. These sampled pigs were considered subsamples within each pen, and the pen remained the experimental unit for subsequent statistical analysis. Blood samples (10 mL) were collected from the jugular vein and allowed to stand for 2–3 h. The blood was centrifuged at 1000 g for 10 min at 4 °C to separate the serum, which was then stored at −20 °C for future use. After a 12 h fasting period, three pigs close to the average body weight were selected for slaughter in each replicate. About 200 g of left longissimus thoracis muscle (commonly known as *longissimus dorsi muscle*) was taken from the 10th to the 13th intercostal space within 45 min after slaughter, and the visible fat and connective tissue in the muscle were carefully removed for subsequent meat quality analysis. At the same time, the contents of jejunum and cecum were collected for intestinal microflora analysis.

### 2.5. Fermented Feed Parameters

Dry matter (DM) content was determined by the oven-drying method (GB/T 6435-2014) [16], the crude protein (CP) content was determined by the Kjeldahl nitrogen determination method (GB/T 6432-2018) [17], the crude fiber (CF) content was determined in accordance with the national standard GB/T 5009.10-2003 [18], the neutral detergent fiber (NDF) content was determined in accordance with the national standard (GT/T 20806-2022) [19], the acid detergent fiber (ADF) content was determined in accordance with the agricultural standard (NY/T 1459-2022) [20], and the soluble sugar (SS), ammonia nitrogen (NH_3_-N), and starch (ST) were determined using the relevant detection kits provided by Shanghai Enzyme-Link Biotechnology Co., Ltd. (Shanghai, China) Lactic acid (LA), acetic acid (AA), propionic acid (PA), and butyric acid (BA) were determined by gas chromatography. The pH value was measured using the portable pH meter manufactured by Thermo Fisher Scientific, Waltham, MA, USA. For each sample, three parallel measurements were performed, and the relative deviation had to fall within the acceptable range. The chemical composition analysis, pH and volatile fatty acid content changes of the fermented feed are shown in Table 2.

### 2.6. Determination of Growth Performance and Carcass Traits

All pigs were individually weighed after a 12 h fasting period on day 0 and day 50 of the formal trial. For each pen, the mean body weight of the six pigs was used to calculate initial body weight (IBW), final body weight (FBW), and average daily gain (ADG). Feed offered and feed refusals were recorded daily on a pen basis. Average daily feed intake and feed-to-gain ratio were calculated for each pen. Average daily gain (ADG, kg/d) was calculated as follows: ADG = (FBW − IBW)/trial period (days). During the feeding period, the daily feed supply and feed refusals were continuously recorded on a pen basis. The average daily feed intake (ADFI, kg/head/d) was calculated as follows: ADFI = total feed intake (kg)/number of days/number of pigs per pen. The feed-to-gain ratio (F/G) was calculated as the total feed intake per pen divided by the total body weight gain per pen. For carcass traits and meat quality measurements, three pigs with body weights close to the pen average were selected from each pen. The values obtained from the three pigs within the same pen were averaged, and this pen-level mean was used for statistical analysis. Carcass weight and dressing percentage were determined according to NY/T 825-2004 [21], Technical Specification for the Determination of Carcass Traits in Lean-Type Pigs. Average backfat thickness was calculated from measurements taken at the first rib, last rib, and last lumbar vertebra using a vernier caliper.

### 2.7. Determination of Longissimus Dorsi Muscle Quality

*Longissimus dorsi muscle* samples were collected from the three pigs per pen whose body weights were closest to the pen average. The left longissimus thoracis et lumborum muscle samples collected between the 10th and 13th ribs were used for meat quality determination. The measurements of pH at 45 min and 24 h postmortem, lightness (L*), redness (a*), yellowness (b*), water loss rate, cooked meat rate, and shear force were conducted in accordance with NY/T 2793-2015 [22], Objective Evaluation Methods for the Edible Quality of Meat.

### 2.8. Determination of Serum Immune, Serum Biochemical and Antioxidant Indicators

For serum biochemical, antioxidant, and immune indices, serum samples were collected from the three pigs per pen whose body weights were closest to the pen average. These samples were analysed separately for each pen. The mean value of the three pigs within the same pen was then calculated and used as one experimental replicate for statistical analysis. The serum total protein (TP), glucose (GLU), total cholesterol (TC) and triglyceride (TG) were directly measured using a veterinary biochemical analyzer. The contents of superoxide dismutase (SOD), malondialdehyde (MDA), catalase (CAT), and total antioxidant capacity (T-AOC) were determined using the relevant reagent kits provided by Shanghai Enzyme-Linked Biotechnology Co., Ltd. (Shanghai, China). The contents of serum immunoglobulin A (IgA), immunoglobulin M (IgM), immunoglobulin G (IgG), interleukin-2 (IL-2), interleukin-6 (IL-6), interleukin-10 (IL-10), and tumor necrosis factor-α (TNF-α) were determined using the ELISA kit provided by Shanghai Enzyme-Linked Biotechnology Co., Ltd. (Shanghai, China).

### 2.9. Analysis of Intestinal Microorganisms

At the end of the feeding trial, six pigs per treatment were selected for intestinal microbiota analysis from the four replicate pens, with one or two pigs selected from each pen to ensure representation of all pens. The selected pigs had body weights close to the mean body weight of their respective pens. After slaughter, the abdominal cavity was opened under aseptic conditions, and the jejunum and cecum were carefully isolated. Jejunal and cecal contents were collected separately from each pig using sterile instruments, immediately transferred into sterile centrifuge tubes, frozen in liquid nitrogen, and stored at −80 °C until further analysis. The samples were not pooled before DNA extraction or sequencing. Jejunal and cecal contents collected from the three groups of fattening pigs were sent to Shenzhen Microbial Alliance Biotechnology Co., Ltd. (Shen, Guangdong, China) for 16S rRNA gene sequencing analysis. Sequencing was performed using the illumina NovaSeq sequencing platform [23] to analyze the abundance and diversity of the gut microbiota in the jejunum and cecum. Following barcode demultiplexing [24], the raw reads were processed with the QIIME 2 (2023.9) workflow [25] and the DADA2 plugin [26] for quality filtering, denoising, merging, and chimera removal, yielding a high-quality amplicon sequence variant (ASV) table and representative sequences [27,28]. Representative sequences were selected and compared with the SILVA database Chuvochina et al. [29] to obtain species annotation information (using the default qiime feature-classifier classify-sklearn method) [30,31]. All analyses of the microbiome data were performed on the free online platform of the Wekemo Bioincloud cloud system (https://www.bioincloud.tech, accessed on 13 April 2026).

### 2.10. Statistical Analysis

Data were organized using Microsoft Excel (version 2019; Microsoft, Inc., Redmond, WA, USA) and analyzed using SPSS statistical software (version 22.0; SPSS Inc., Chicago, IL, USA). The experiment was conducted as a completely randomized design, with dietary treatment considered as the fixed effect. Before statistical analysis, all data were tested for normality and homogeneity of variance using the Shapiro–Wilk test and Levene’s test, respectively. Data that met the assumptions of normal distribution and homogeneity of variance were analyzed using one-way analysis of variance. When significant treatment effects were detected, Duncan’s multiple range test was used to compare differences among treatment means. Data that did not meet the assumptions of normality or homogeneity of variance were analyzed using the Kruskal–Wallis test. When the Kruskal–Wallis test indicated a significant difference, Dunn’s post hoc test was used for pairwise comparisons among treatments. Results are presented as mean ± standard deviation. Statistical significance was indicated as follows: *p* < 0.05 (*), *p* < 0.01 (**), and *p* < 0.001 (***).

The pen was considered the experimental unit for statistical analysis. For growth performance, average daily feed intake, and feed-to-gain ratio, data were calculated on a pen basis. For carcass traits, meat quality, serum biochemical indices, antioxidant indices, immune indices, and data obtained from pigs within the same pen were averaged before analysis. Because feed intake was recorded at the pen level and only four replicate pens were included per treatment, F/G was interpreted cautiously as a pen-level indicator of feed utilization. For intestinal microbiota analysis, each individual sequenced sample was considered one biological sample. Six jejunal samples and six cecal samples were analyzed per treatment group. Alpha diversity, beta diversity, microbial composition, and differential taxa analyses were performed based on these individual sequencing samples.

## 3. Results

### 3.1. Growth Performance and Carcass Traits

As shown in Table 3, fermented feed supplementation had no significant effect on initial body weight or average daily feed intake (ADFI) in fattening pigs (*p* = 0.825 and *p* = 0.821, respectively). However, significant differences were observed in final body weight, average daily gain (ADG), feed-to-gain ratio (F/G), carcass weight, and average backfat thickness. YR1 showed the highest final body weight and ADG, followed by YR2, whereas CK had the lowest values (*p* < 0.001). The F/G was significantly lower in YR1 and YR2 than in CK (*p* < 0.001), indicating improved feed utilization in the fermented feed groups. However, this result should be interpreted cautiously because F/G was calculated from pen-level feed intake. Carcass weight was also affected by treatment (*p* = 0.008), with YR1 showing a higher value than CK and YR2, while no significant difference was observed between CK and YR2. Dressing percentage did not differ significantly among the three groups (*p* = 0.636). In addition, average backfat thickness differed significantly among treatments (*p* = 0.013), with YR2 showing a lower value than CK, whereas YR1 did not differ significantly from either CK or YR2.

### 3.2. Meat Quality

The effects of fermented feed on meat quality in fattening pigs are presented in Table 4. Dietary fermented feed supplementation had no significant effect on meat lightness (L*), yellowness (b*), pH _45min_, or cooked meat rate (*p* = 0.572, *p* = 0.113, *p* = 0.208 and *p* = 0.459, respectively). However, meat redness (a*) was significantly higher in YR1 and YR2 than in CK (*p* < 0.001), with no significant difference between the two fermented feed groups. The pH _24h_ differed significantly among treatments (*p* = 0.021), with YR1 showing a lower value than CK and YR2. Shear force was significantly reduced in YR1 and YR2 compared with CK (*p* = 0.020), whereas no difference was observed between YR1 and YR2. In addition, water loss rate was significantly affected by treatment (*p* = 0.041), with YR1 showing a lower value than CK and YR2, while YR2 did not differ from CK.

### 3.3. Serum Biochemical Indices

As shown in Table 5, no significant differences were observed in serum Glu, TC, TG, or TP concentrations among CK, YR1, and YR2 (*p* = 0.836, *p* = 0.293, *p* = 0.975, and *p* = 0.136, respectively), indicating that the fermented feeds had no marked effect on the measured serum biochemical indices under the conditions of this study.

### 3.4. Serum Antioxidant Capacity

As shown in Table 6, dietary treatment affected serum SOD activity, with YR1 and YR2 showing higher values than CK (*p* = 0.020). No significant differences were observed in serum MDA, CAT, or T-AOC levels among CK, YR1, and YR2 (*p* = 0.211, *p* = 0.112, and *p* = 0.899, respectively).

### 3.5. Serum Immune Status

Table 7 shows that serum IgA and IgM concentrations were not affected by dietary treatment (*p* = 0.257 and *p* = 0.220, respectively). Serum IgG concentration was higher in YR2 than in CK and YR1 (*p* = 0.024). A similar pattern was observed for IL-2, with YR2 showing a higher value than the other two groups (*p* = 0.014). Serum IL-6 concentration did not differ among CK, YR1, and YR2 (*p* = 0.845). In contrast, serum IL-10 concentration was higher in YR1 and YR2 than in CK (*p* = 0.016). Serum TNF-α concentration was lower in YR1 than in CK and YR2 (*p* = 0.026).

### 3.6. 16S rRNA Sequencing Analysis of the Effects of Bacillus Fermented Feed on the Intestinal Microflora of Fattening Pigs

#### 3.6.1. Overview of Sequencing Data

**Figure 1:**
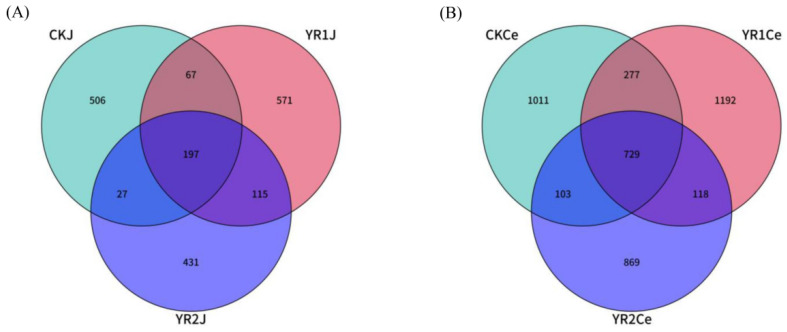
Venn diagram of ASV distribution between different groups. (**A**) Jejunum; (**B**) cecum.

Based on the paired-end raw sequencing data generated by the Illumina NovaSeq platform, high-quality non-chimeric amplicon sequence variants (ASVs) were obtained after demultiplexing, quality filtering, denoising, merging, and chimera removal. Venn diagrams were used to visualize the distribution of shared and group-specific ASVs among treatments. In the jejunal samples (Figure 1A), the CK, YR1, and YR2 groups contained 797, 950, and 770 ASVs, respectively, with 197 ASVs shared among the three groups. In the cecal samples (Figure 1B), the CK, YR1, and YR2 groups contained 2120, 2316, and 1819 ASVs, respectively, with 729 ASVs shared among the three groups. These results indicate that the three groups differed in the distribution of shared and unique ASVs. These results show differences in shared and unique ASV distributions among groups, but Venn diagrams are descriptive and do not indicate significant changes in overall microbial diversity or community structure.

#### 3.6.2. Alpha Diversity Analysis

**Figure 2:**
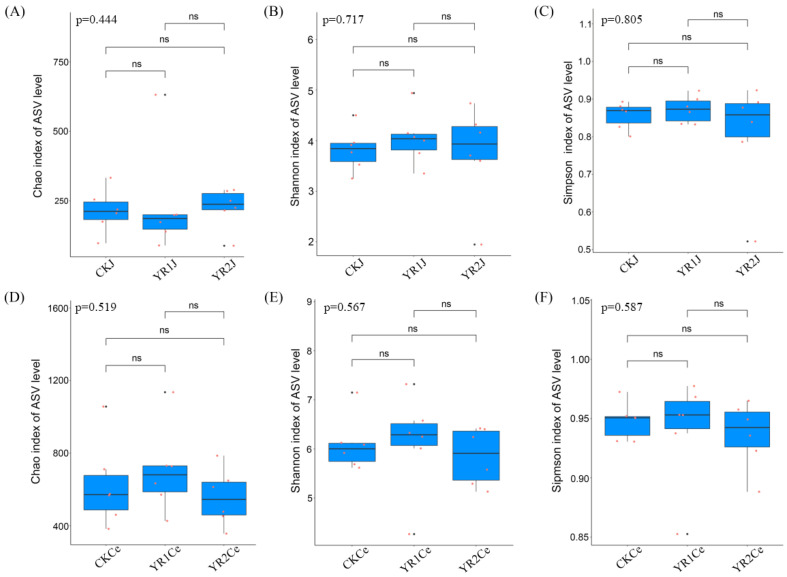
Alpha diversity analysis of microorganisms in jejunum and cecal contents. (**A**,**D**) Multiple comparison of Chao1 index between groups; (**B**,**E**) multiple comparison of Shannon index between groups; (**C**,**F**) multiple comparisons of Simpson index among groups. Red points represent individual biological samples. The blue boxes indicate the interquartile range, the horizontal line within each box represents the median, and the whiskers extend to 1.5 times the interquartile range. Black points outside the whiskers indicate potential outliers.

The Chao1, Shannon, and Simpson indices were used to evaluate microbial richness and diversity. As shown in Figure 2, no significant differences in alpha diversity were observed among the three groups in either the jejunum or cecum. In the jejunum, the Chao1, Shannon, and Simpson indices did not differ among treatments (*p* = 0.444, *p* = 0.717, and *p* = 0.805, respectively). Similarly, no significant differences were detected in the cecum for the Chao1, Shannon, or Simpson indices (*p* = 0.519, *p* = 0.567, and *p* = 0.587, respectively). These results suggest that, under the conditions of this study, dietary *Bacillus*-fermented feed did not markedly alter the richness or diversity of the jejunal and cecal microbial communities.

#### 3.6.3. Beta Diversity Analysis

**Figure 3:**
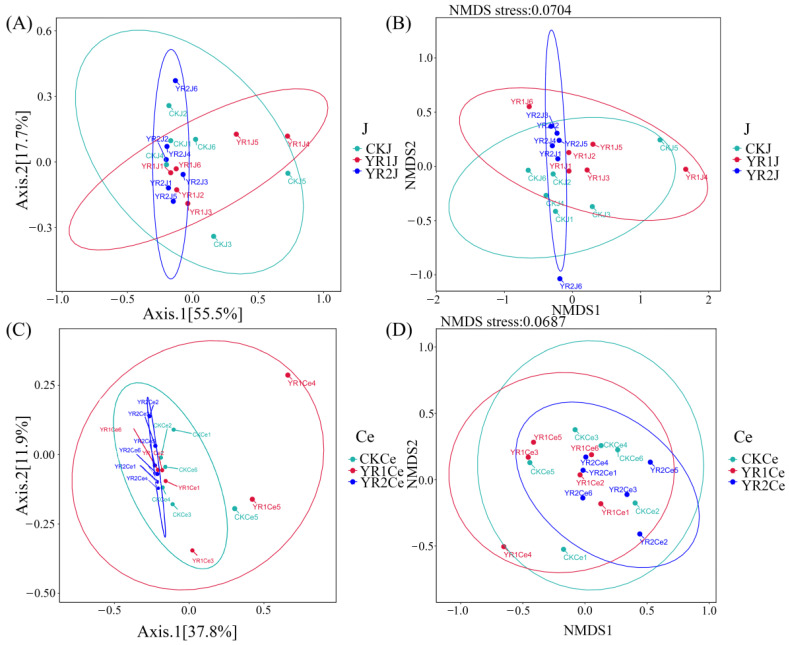
Beta diversity analysis of microorganisms in jejunum and cecal contents. (**A**,**C**) Principal coordinate analysis (PCoA); (**B**,**D**) nonmetric multidimensional scaling (NMDS). Different colored circles represent individual samples from different treatment groups. The colored ellipses indicate the distribution range of samples within each treatment group.

Principal coordinate analysis (PCoA) based on Bray–Curtis dissimilarity and non-metric multidimensional scaling (NMDS) were used to evaluate differences in microbial community structure among the three groups. As shown in (Figure 3A,B), the jejunal samples from CKJ, YR1J, and YR2J showed partial overlap, and no clear separation was observed among the three groups. PERMANOVA analysis further showed that the overall microbial community structure of the jejunum did not differ significantly among treatments (*p* = 0.218).

A similar pattern was observed in the cecal samples. The PCoA and NMDS plots showed that CKCe, YR1Ce, and YR2Ce samples were partially overlapped, without distinct separation among the three groups (Figure 3C,D). PERMANOVA analysis also showed no significant difference in the overall microbial community structure of the cecum among treatments (*p* = 0.078). These results indicate that dietary *Bacillus*-fermented feed did not markedly alter the overall microbial community structure in either the jejunum or cecum.

#### 3.6.4. Effects of Fermented Feed Treatments with Different Bacillus Strains on the Microbial Community Composition at the Phylum and Genus Levels

**Figure 4:**
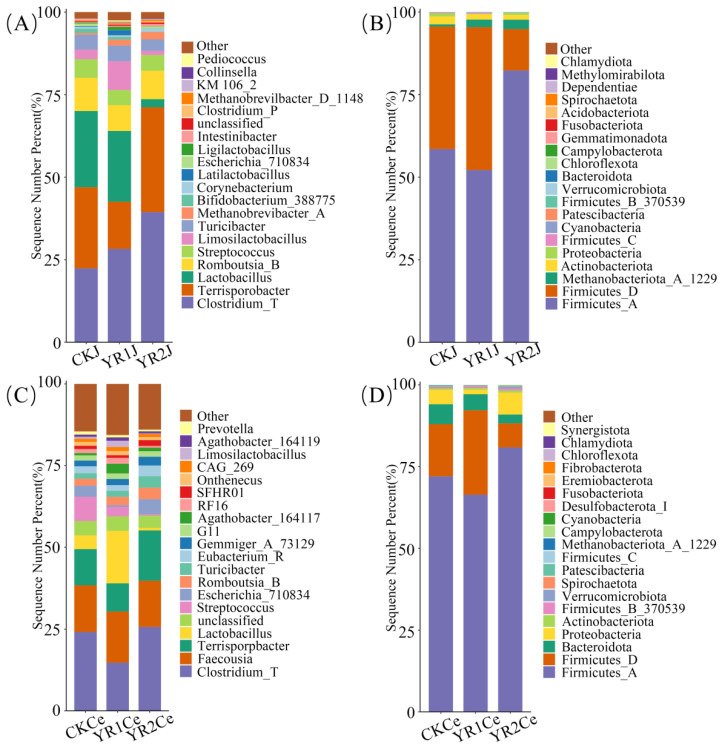
Abundance diagram of intestinal microbial flora structure of fattening pigs. (**A**) Genus-level composition in the jejunum; (**B**) phylum-level composition in the jejunum; (**C**) genus-level composition in the cecum; (**D**) phylum-level composition in the cecum. Analysis of Microbial Species Differences in the Jejunum and Cecum.

The microbial composition was analyzed at the genus level (Figure 4A,C). In the jejunal microbiota, the three groups of intestinal microbial genera with higher abundance include *Clostridium_T*, *Terrisporobacter*, *Lactobacillus*, *Romboutsia_B*, *Streptococcus*, *Limosilactobacillus*, *Turicibacter*, *Methanobrevibacter_A*, *Bifidobacterium_388775*, and *Corynebacterium*. In the CKJ and YR1J groups, the three most abundant taxa were *Clostridium_T* (22.37% and 28.28%), *Terrisporobacter* (24.58% and 14.26%), and *Lactobacillus* (23.09% and 21.44%). In the YR2J group, the top three taxa were *Clostridium_T* (39.39%), *Terrisporobacter* (31.76%), and *Romboutsia_B* (8.64%). Compared with the control group, the relative abundance of *Clostridium_T* increased in both treatment groups, while that of *Lactobacillus* decreased.

In the cecal microbiota, the three groups of *bacteria* with higher abundance included *Clostridium_T*, *Faecousia*, *Terrisporobacter*, *Lactobacillus*, *Streptococcus*, *Escherichia_710834*, and *Romboutsia_B*. In the CKCe and YR2Ce groups, the top three taxa were *Clostridium_T* (24.14% and 25.68%), *Faecousia* (14.27% and 14.12%), and *Terrisporobacter* (11.01% and 15.35%). In the YR1Ce group, the top three taxa were *Clostridium_T* (14.74%), *Faecousia* (15.66%), and *Lactobacillus* (15.99%).

We analyzed the microbial composition at the phylum level (Figure 4B,D). In the jejunal microbiota, the three groups of *bacteria* with higher abundance included *Firmicutes_A*, *Firmicutes_D*, *Methanobacteriota_A_1229*, *Proteobacteria*, *Actinobacteriota*, *Firmicutes_C*, *Cyanobacteria*, *Patescibacteria*, *Firmicutes_B_370539*, and *Verrucomicrobiota*. In the CKJ group, the three most abundant taxa were *Firmicutes_A* (58.50%), *Firmicutes_D* (37.08%), and *Actinobacteriota* (2.35%). In the two treatment groups, the three most abundant taxa were *Firmicutes_A* (52.16% and 82.29%), *Firmicutes_D* (43.27% and 12.52%), and *Methanobacteriota_A_1229* (2.26% and 2.85%). All three groups exhibited a community structure characterized by *Firmicutes_A* as the dominant phylum, reflecting the relative stability of the jejunal microbial composition.

In the cecal microbiota, the three groups of bacteria with higher abundance included *Firmicutes_A*, *Firmicutes_D*, *Bacteroidota*, *Proteobacteria*, *Actinobacteriota*, *Firmicutes_B_370539*, *Verrucomicrobiota*, *Spirochaetota*, *Patescibacteria*, and *Firmicutes_C*. In the CKCe and YR1Ce groups, the top three taxa were *Firmicutes_A* (72.01% and 66.39%), *Firmicutes_D* (15.98% and 25.84%), and *Bacteroidota* (6.09% and 4.90%). In the YR2Ce group, the top three taxa were *Firmicutes_A* (80.84%), *Firmicutes_D* (7.38%), and *Proteobacteria* (6.70%). At the phylum level, *Firmicutes_A* was the dominant taxon in both the jejunum and cecum across the three groups. The abundance differences in phyla such as *Bacteroidota* and *Verrucomicrobiota* indicated treatment-specific modulation of the cecal microbial community.

**Figure 5:**
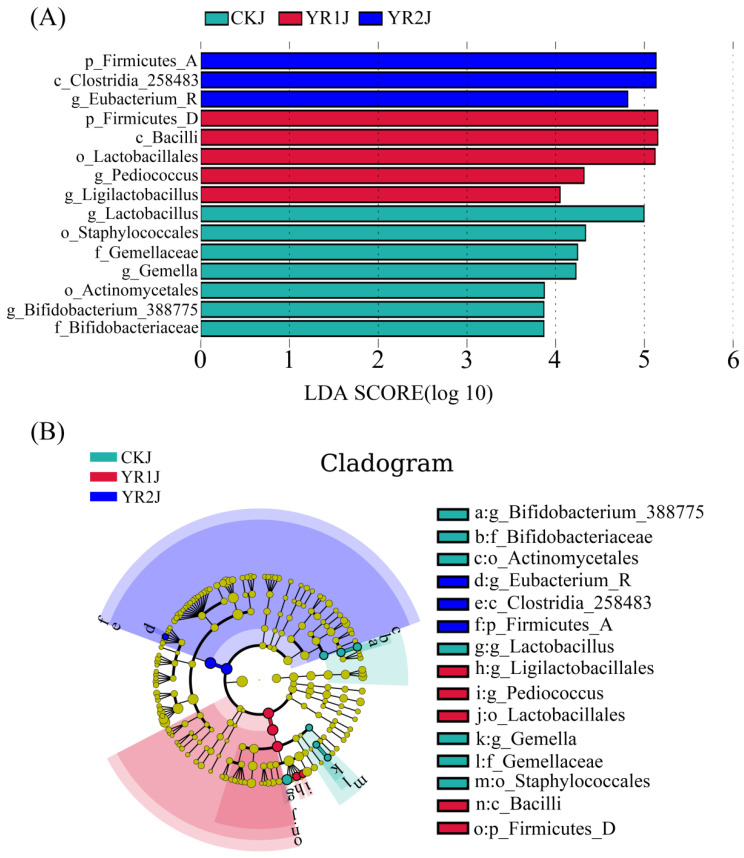
Lefse analysis of the jejunal microbiota. (**A**) LDA score histogram; (**B**) LEfSe cladogram. In the cladogram, cyan, red, and blue nodes and branches represent taxa significantly enriched in the CKJ, YR1J, and YR2J groups, respectively, whereas yellow nodes indicate taxa without significant differences among groups. The concentric circles from the center outward represent the phylum, class, order, family, and genus levels, respectively, and the node size reflects the relative abundance of each taxon.

**Figure 6:**
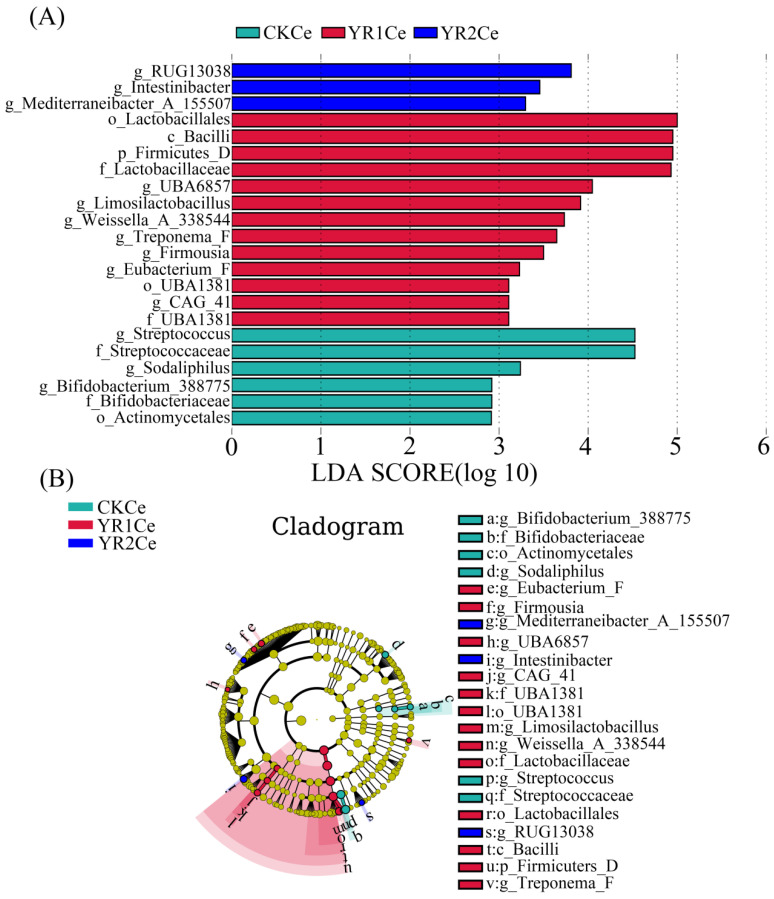
Lefse analysis of the cecal microbiota. (**A**) LDA score histogram; (**B**) LEfSe cladogram. In the cladogram, cyan, red, and blue nodes and branches represent taxa significantly enriched in the CKCe, YR1Ce, and YR2Ce groups, respectively, whereas yellow nodes indicate taxa without significant differences among groups. The concentric circles from the center outward represent the phylum, class, order, family, and genus levels, respectively, and the node size reflects the relative abundance of each taxon.

The Lefse analysis of microbial groups indicated that both types of spore-forming bacteria-based fermented feed significantly affected the abundance of specific groups (Figure 5 and Figure 6). In this study, differences in the effects of each group on gut microbial species abundance were compared, and differential taxa were identified based on LDA effect size and *p* value thresholds (LDA score ≥ 4, *p* < 0.05). At the genus level, 12 and 7 specific taxa were identified in the jejunum and cecum, respectively, with 4, 5, and 3 significantly enriched in the CKJ, YR1J, and YR2J groups, respectively. The most significantly enriched taxon in the YR1J and YR2J groups were *Firmicutes_A* and *Firmicutes_D*, respectively. The CKCe and YR1Ce groups showed significant enrichment of 2 and 5 genera, respectively, whereas no differential taxa were identified in the YR2Ce group. The most significantly enriched taxon in the YR1Ce group was *Lactobacillaceae*.

#### 3.6.5. Phylogenetic Analysis of Microbiota in the Jejunum and Cecum

**Figure 7:**
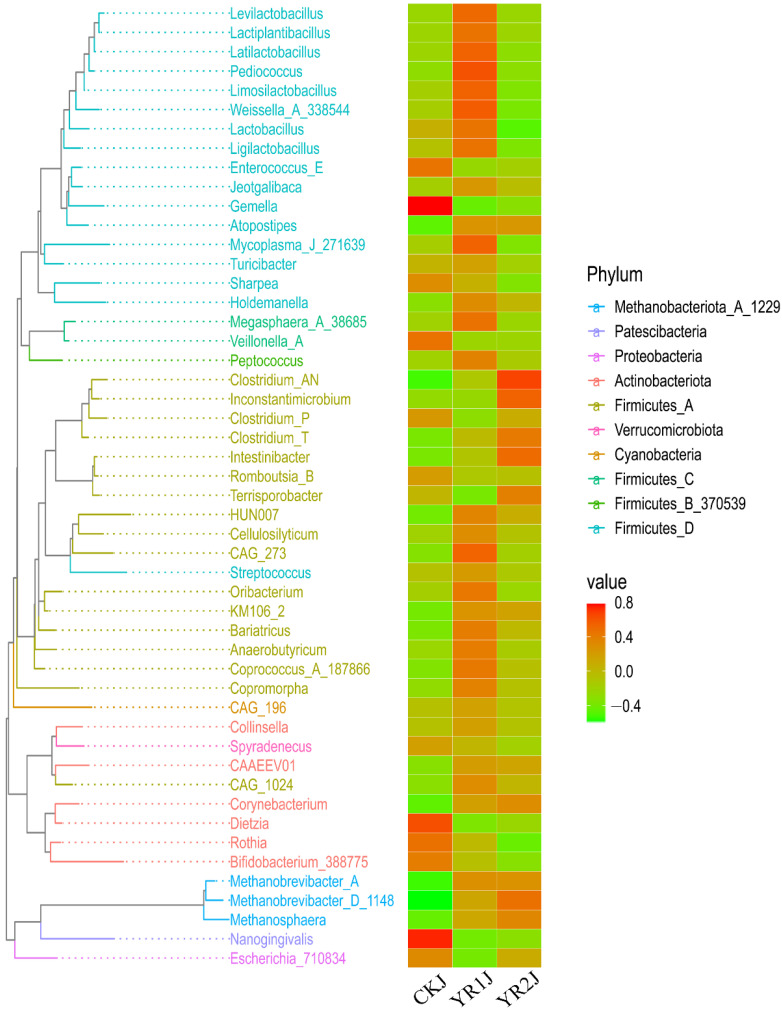
Phylogenetic tree and inter-group abundance distribution heatmap of the jejunal microbiota.

**Figure 8:**
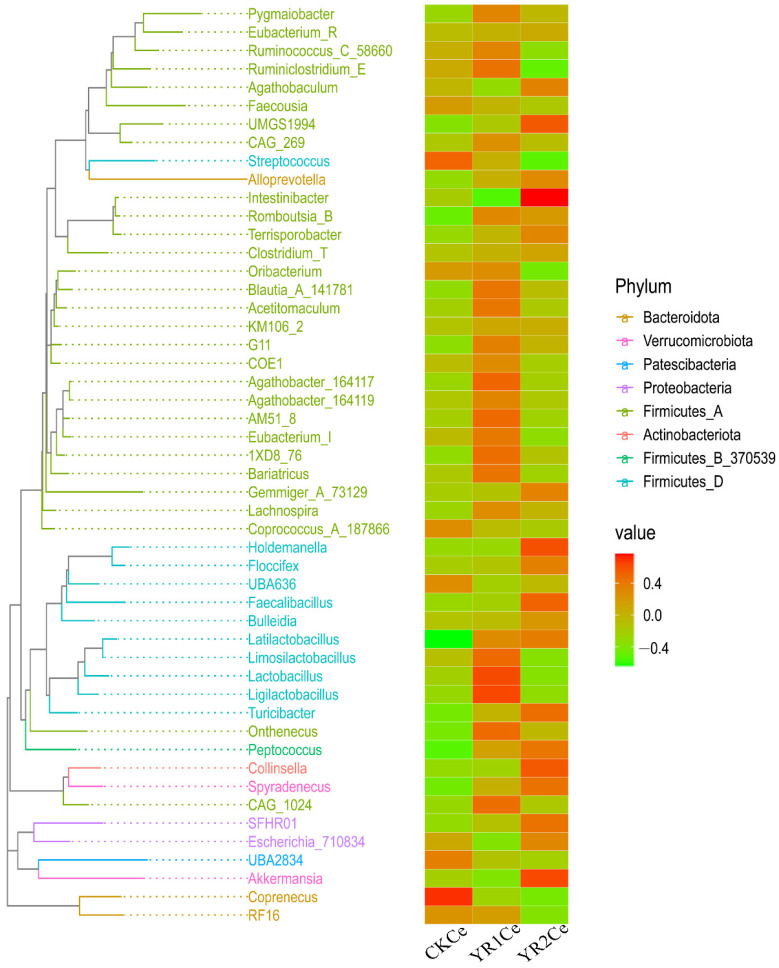
Phylogenetic tree and inter-group abundance distribution heatmap of the cecal microbiota.

Representative ASV sequences were used for phylogenetic analysis, and the evolutionary tree and absolute abundance were visualized in a heatmap (Figure 7 and Figure 8). The core microbial communities of the jejunum and cecum are different at the genus level. The jejunal microbiota was mainly distributed among *Firmicutes*, *Actinobacteriota*, *Proteobacteria*, and *Methanobacteriota*, indicating that the jejunal microbial community exhibited clear taxonomic clustering characteristics. Moreover, obvious differences were observed between the CKJ group and the treatment group in microbial composition and relative abundance. Compared with the control group, in the YR1J and YR2J groups, the lactic acid bacteria-related flora and some short-chain fatty acid-metabolizing bacteria showed an enrichment trend, while some potential opportunistic pathogenic bacteria and methane-producing-related bacteria were relatively reduced, suggesting that both strains of *Bacillus* can regulate the microecological structure of the jejunum.

The cecal microbiota was primarily composed of *Firmicutes*, *Bacteroidota*, *Actinobacteriota*, *Proteobacteria*, *Verrucomicrobiota*, and *Patescibacteria*, among which Firmicutes was the predominant phylum. Compared with the CKCe group, in the treatment group, various bacterial communities related to fermentation metabolism and short-chain fatty acid production (such as *Lactobacillus*, *Limosilactobacillus*, *Blautia*, *Coprococcus*, *Lachnospira*, *Ruminococcus*, etc.) showed varying degrees of enrichment, and the abundance of some potential opportunistic pathogens (such as *Escherichia*) also changed.

## 4. Discussion

### 4.1. Fermented Feed Quality

In the present study, the crude protein contents of the YR1 and YR2 groups reached 16.93% and 16.32%, respectively, after fermentation with *Bacillus amyloliquefaciens* YR3.2 and *Bacillus licheniformis* YR3.1, both of which were significantly higher than that of the CK group (14.29%). This increase may be attributed to the strong nitrogen conversion capacity of probiotic *Bacillus* strains. During fermentation, *Bacillus* species can secrete extracellular proteases, which promote the degradation and transformation of nitrogen-containing substrates and facilitate the formation of bacterial biomass protein [32,33,34,35]. Notably, the crude protein content was higher in the YR1 group than in the YR2 group, suggesting that *Bacillus amyloliquefaciens* YR3.2 may have a greater ability to convert nitrogen sources or degrade non-protein components in the tested feed substrate than *Bacillus licheniformis* YR3.1. These findings further indicate that the effects of different *Bacillus* strains on the nutritional composition of fermented feed are strain-specific.

Regarding structural carbohydrates, fermentation in all treated groups significantly reduced the contents of CF and ADF. Among them, the decrease in ADF was most pronounced in the feed fermented with *Bacillus licheniformis* YR3.1, which may be attributed to the relatively high cellulase and xylanase activities produced by this strain [36]. The NDF content also showed a decreasing trend, which is consistent with the findings of Qi et al. [37], who reported that solid-state fermentation with *Bacillus licheniformis* significantly reduced NDF and ADF contents in soybean meal. These results suggest that fermentation effectively disrupted the plant cell wall structure, which may facilitate nutrient release in the digestive tract and improve feed utilization efficiency [38].

Regarding nitrogen metabolism, the NH_3_-N content in the YR1 group was only 24.41% of that in the CK group. This result is consistent with the mechanisms described by Krizsan et al. [39], who suggested that fermentation can improve feed digestibility by enhancing nitrogen utilization. The markedly lower NH_3_-N content in the YR1 group indicates that *Bacillus amyloliquefaciens* YR3.2 may more efficiently assimilate free ammonia into microbial protein and reduce nitrogen loss during fermentation. This difference may be related to the urease activity and nitrogen assimilation efficiency of this strain [40,41].

The carbon source utilization characteristics show that the fermentation treatment significantly reduced the SS content, while the ST content significantly increased to 201.56–203.64 mg/g, indicating that *Bacillus* preferentially utilized soluble sugars as a carbon source, and possibly released bound starch through enzymatic action or promoted the synthesis of extracellular polysaccharides [42,43]. Both types of bacilli can improve feed quality. The *Bacillus amyloliquefaciens* strain performs better in increasing CP, reducing ADF and NH_3_-N.

### 4.2. Growth Performance and Carcass Performance

In the present study, feeds fermented with *Bacillus amyloliquefaciens* YR3.2 and *Bacillus licheniformis* YR3.1 increased the average daily gain and reduced the feed-to-gain ratio (F/G) of finishing pigs, indicating improved feed conversion efficiency. However, because feed intake was measured on a pen basis and only four replicate pens were included per treatment, the F/G results should be interpreted as pen-level observations and with appropriate caution. Zhang et al. [44] found that dietary supplementation with 4% fermented rice wine lees co-fermented by *Bacillus subtilis* and *Enterococcus faecium* had no significant effects on average daily feed intake or average daily gain in finishing pigs with an initial body weight of 89.59 ± 1.30 kg after a 50-day feeding period. The discrepancy between these findings may be attributed to several factors, including differences in diet composition, feed form, fermentation substrate, bacterial strains, inclusion level, and potential interactions with other feed additives.

### 4.3. Meat Quality, pH Value and Shearing Force

The results of this study showed that although there was no significant difference in lightness (L*) and yellowness (b*) between the control group and the treatment groups, the redness values (a*) in the treatment groups were significantly increased by 62.05% and 55.47%, respectively, compared with the control group. This finding is consistent with the conclusions of Ding et al. [45], who reported that fermented feed regulates the oxidation state of myoglobin by enhancing antioxidant capacity. Organic acids produced during feed fermentation, such as lactic acid and acetic acid, may promote iron absorption by lowering intestinal pH, thereby increasing myoglobin content [46].

As a key parameter to characterize the degree of muscle glycolysis and meat quality characteristics after slaughter, the dynamic change of pH value directly affects the water retention and tenderness of meat [47,48]. This study found that the pH value at 45 min postmortem did not differ significantly in the treatment group, indicating that fermented feed had no significant regulatory effect on the initial postmortem glycolytic rate; however, at 24 h, the pH value in the YR1 group was significantly lower than that in the control group, and this difference may be associated with the microbial composition and metabolic characteristics of different fermented feeds [49,50].

In the present study, the shear force values in the YR1 and YR2 groups decreased by 19.06% and 12.02%, respectively, compared with the CK group, indicating that both *Bacillus*-fermented feeds improved pork tenderness. In addition, the water loss rate in the YR1 group was significantly reduced by 16.21% compared with the CK group, suggesting that feed fermented with *Bacillus amyloliquefaciens* YR3.2 was more effective in improving the water-holding capacity of pork. Lower water loss may help maintain muscle juiciness and structural integrity, thereby contributing to reduced shear force. These improvements may be associated with the enhanced nutritional quality of fermented feed and the strain-specific enzymatic activity of *Bacillus*, which could influence muscle protein metabolism and postmortem proteolysis [51,52]. Overall, these results suggest that fermented feed can improve meat quality in finishing pigs, particularly in terms of tenderness and water-holding capacity, with the YR1 group showing a more comprehensive improvement than the YR2 group.

### 4.4. Serum Biochemical, Serum Immune and Antioxidant Indexes

In the present study, serum TP levels were numerically higher in the YR1 and YR2 groups than in the CK group, although the differences were not statistically significant. This tendency may be related to the ability of probiotic *Bacillus* strains to secrete alkaline and neutral proteases, which can hydrolyze complex dietary proteins into small peptides and amino acids, thereby potentially improving intestinal protein digestion and absorption [53]. No significant differences were observed in serum GLU, TC, TG, or TP concentrations among the three groups, suggesting that dietary *Bacillus*-fermented feed did not markedly disturb serum biochemical homeostasis or lipid metabolism under the conditions of this study. Similar results were reported by Fu et al. [54] and Saleh et al. [55], who found that fermented feed had no obvious effect on serum biochemical or lipid metabolism-related parameters in poultry. However, this finding partly differs from that of Czech et al. [56], who reported that dietary fermented rapeseed meal (FRSM) modulated several blood biochemical parameters in sows and piglets. The inconsistent responses may be related to differences in animal physiological stage, fermented substrate, dietary inclusion level, and feeding duration.

Antioxidant capacity is important for maintaining the health and production performance of fattening pigs, as it helps protect the body from oxidative stress damage [57]. In the present study, serum SOD activity was significantly higher in the YR1 and YR2 groups than in the CK group, suggesting that both *Bacillus*-fermented feeds enhanced the antioxidant defense capacity of fattening pigs. SOD is a key antioxidant enzyme responsible for scavenging superoxide anion radicals, and its increased activity may be associated with bioactive metabolites produced during fermentation, such as antioxidant peptides and organic acids, which could contribute to the activation of host antioxidant responses [58]. Although there was no significant difference in CAT, MDA and T-AOC in the YR1 group among the three groups, CAT showed an increasing trend. The improvement of antioxidant status induced by fermented feed is mainly reflected in the increase of SOD activity rather than the extensive changes of all antioxidant indexes.

In the present study, dietary *Bacillus*-fermented feed affected several serum immune and inflammatory parameters in fattening pigs. Notably, serum IL-10 concentrations were significantly higher in both the YR1 and YR2 groups than in the CK group, whereas IL-6 did not differ among treatments. In addition, TNF-α was significantly lower in the YR1 group than in the CK and YR2 groups. IL-10 is an important anti-inflammatory cytokine that limits excessive inflammatory responses and contributes to immune homeostasis [59]. Therefore, the increase in IL-10, together with the unchanged IL-6 level and the reduced TNF-α concentration in the YR1 group, suggests that *Bacillus*-fermented feed may promote a more balanced anti-inflammatory immune status rather than inducing systemic inflammation. This response may be partly related to the improved fermentation quality of the diet, including increased organic acid production and reduced ammonia nitrogen, which could contribute to a more favorable intestinal environment and reduce inflammatory stimulation from the gut [60].

These findings are relevant to the current understanding of swine inflammation and necrosis syndrome (SINS) and gut-health-related systemic inflammation. Previous studies have suggested that feed composition, intestinal microbiota, gut barrier integrity, and microbe-associated molecular patterns are closely involved in systemic inflammatory responses in pigs [61]. When intestinal barrier function is impaired, microbial components such as lipopolysaccharides may enter the circulation and activate inflammatory pathways, thereby increasing the risk of inflammation-associated disorders and compromising animal welfare. In this context, the higher IL-10 concentrations observed in the YR1 and YR2 groups may indicate an enhanced anti-inflammatory regulatory capacity, while the lower TNF-α level in the YR1 group suggests a stronger potential to alleviate systemic inflammatory pressure [62]. From an animal health and welfare perspective, these changes may be beneficial because excessive or persistent inflammation is associated with reduced resilience, impaired gut function, and welfare-related problems such as inflammatory lesions and tail damage. However, because SINS lesions, intestinal barrier markers, and circulating endotoxin levels were not directly measured in this study, the present results should be interpreted as supportive evidence that *Bacillus*-fermented feed may improve immune balance and systemic health status, rather than as direct evidence for the prevention of SINS.

### 4.5. Jejunal and Cecal Microbiota

The gut microbiota plays an important role in host nutrient metabolism, including carbohydrate, amino acid, and lipid metabolism, and its composition and metabolic capacity vary among different intestinal segments [63]. Ji et al. [64] reported that fermented liquid feed containing a probiotic consortium did not significantly affect alpha diversity in pigs. In the present study, the Venn diagram showed differences in the distribution of shared and unique ASVs among the CK, YR1, and YR2 groups, whereas alpha and beta diversity analyses revealed no significant differences in overall microbial richness, diversity, or community structure among treatments. These results suggest that dietary *Bacillus*-fermented feed did not markedly disturb the overall microbial community structure in either the jejunum or cecum. Czech et al. [65] reported that dietary FRSM/FSBM improved intestinal microbial composition and intestinal histological structure in weaned piglets. In contrast, no significant changes in overall alpha or beta diversity were observed in the present study, although the relative abundance of several genera changed. This difference may be related to animal physiological stage, fermented substrate, dietary inclusion level, and intestinal segment. Therefore, in fattening pigs, *Bacillus*-fermented complete feed may exert its effects mainly through selective modulation of specific bacterial taxa rather than extensive remodeling of the overall microbial community structure.

At the phylum level, *Firmicutes* was the dominant phylum in both jejunal and cecal samples, with a relative abundance exceeding 80%, which is generally consistent with previous studies on the intestinal microbiota of pigs [66,67]. Other major phyla, including *Actinobacteriota*, *Bacteroidetes*, *Proteobacteria*, and *Methanobacteriota*, have been associated with fiber degradation, carbohydrate utilization, and intestinal fermentation processes [68,69]. At the genus level, the YR1 group showed increased *Clostridium* and decreased *Terrisporobacter* in the jejunum, whereas both genera decreased in the cecum. In contrast, the YR2 group showed increased *Clostridium* and *Terrisporobacter* in the jejunum, and *Terrisporobacter* also increased in the cecum. *Terrisporobacter* and related taxa have been reported to participate in carbohydrate metabolism and short-chain fatty acid production [70]. These results indicate that *Bacillus amyloliquefaciens* YR3.2 and *Bacillus licheniformis* YR3.1 may regulate intestinal microbiota in a strain-specific manner. Overall, *Bacillus*-fermented feed did not significantly alter overall microbial diversity, but it may selectively modulate specific bacterial taxa, thereby contributing to intestinal microecological stability in fattening pigs.

### 4.6. Economic Considerations of Bacillus-Fermented Feed

Based on the national average live pig price in China in the third week of May 2025 (14.93 CNY/kg) and the average price of finishing pig compound feed (3.42 CNY/kg), a preliminary economic estimation was conducted. Since the *Bacillus* strains used in the present study were isolated and preserved in our laboratory, the additional fermentation cost mainly included LB medium components and fermentation-related consumables, as shown in Table 8. The estimated fermentation cost was approximately 134.30 CNY per pig. Before deducting the fermentation cost, the estimated income of the YR1 and YR2 groups increased by 195.19 and 53.44 CNY per head (Table 9), respectively, compared with the CK group. Among the two fermented feed treatments, the YR1 group showed the highest economic benefit, with an additional net return of 60.89 CNY per head after deducting the fermentation cost. This advantage may be mainly associated with its higher final body weight and relatively lower feed intake. However, although the sales income of pigs in the YR2 group was higher than that in the CK group, the increased income was insufficient to cover the fermentation cost, resulting in a negative economic return of −80.86 CNY per head.

These results indicate that, under the conditions of this study, feed fermented with *Bacillus amyloliquefaciens* YR3.2 may have better economic feasibility than feed fermented with *Bacillus licheniformis* YR3.1. Nevertheless, this calculation represents only a preliminary estimation. Future studies should further evaluate the economic value of fermented feed under production-scale fermentation conditions and include more comprehensive cost factors, such as labor, energy consumption, equipment depreciation, storage, transportation, and market price fluctuations.

## 5. Conclusions

This study showed that fermentation *with Bacillus amyloliquefaciens* YR3.2 and *Bacillus licheniformis* YR3.1 improved the nutritional quality of feed to a certain extent. Dietary supplementation with these *Bacillus*-fermented feeds increased the final body weight of finishing pigs and improved some meat quality traits. In addition, *Bacillus*-fermented feed increased serum SOD activity and regulated several immune-related indicators, suggesting potential benefits for antioxidant capacity and immune status [71]. Intestinal microbiota analysis showed that fermented feed induced changes in the relative abundance of specific bacterial taxa, including *Firmicutes*, *Bacteroidetes*, *Terrisporobacter*, *Faecousia*, and *Clostridium_T*, without significantly altering overall intestinal microbial diversity. In summary, feeds fermented with *Bacillus amyloliquefaciens* YR3.2 and *Bacillus licheniformis* YR3.1 may contribute to improvements in growth performance, meat quality, antioxidant status, and intestinal microecological stability in Duroc × Landrace × Large White finishing pigs. These findings provide a theoretical basis and experimental reference for the application of probiotic *Bacillus*-fermented feed in pig production.

## Figures and Tables

**Table 1:** Ingredient composition and nutritional level of basal diet (DM level).

Ingredient (%)	Content	Nutrient Levels	Content
Corn	71.00	DE, MJ/kg	13.46
Soybean meal	13.00	CP, %	14.28
Bran	8.00	CF, %	20.18
Premix ^1^	8.00	Ca, %	0.72
		P, %	0.53
		Lys, %	0.96
		Trp, %	0.13

^1^ The premix provided the following nutrients per kilogram of diet: vitamin A, 44,000 IU; vitamin D3, 7000 IU; vitamin E, 200 mg; vitamin K3, 15 mg; vitamin B2, 35 mg; and pantothenic acid, 88 mg. The mineral elements were copper, 120 mg; iron, 770 mg; zinc, 280 mg; manganese, 300 mg; iodine, 5 mg; and selenium, 2 mg.

**Table 2:** Changes in Nutrient Composition, pH, and Volatile Fatty Acids in Fermented Feed.

Item	CK ^1^	Treatment	
		YR1	YR2
DM, %	43.62 ± 1.12	44.22 ± 1.83	46.58 ± 1.72
CP, %	14.29 ± 0.12 ^b^	16.93 ± 0.45 ^a^	16.32 ± 0.25 ^a^
CF, %	20.18 ± 0.13 ^a^	17.00 ± 0.47 ^c^	17.61 ± 0.08 ^b^
NDF, %	48.85 ± 1.56	44.90 ± 1.75	45.16 ± 1.85
ADF, %	23.18 ± 0.22 ^a^	18.91 ± 0.22 ^b^	17.41 ± 0.22 ^c^
NH_3_-N, μg/g	5971.52 ± 31.75 ^a^	1457.73 ± 40.72 ^c^	2855.78 ± 60.68 ^b^
SS, mg/g	16.17 ± 0.18 ^a^	9.33 ± 0.23 ^c^	10.60 ± 0.46 ^b^
ST, mg/g	144.91 ± 2.25 ^b^	201.56 ± 5.80 ^a^	203.64 ± 6.85 ^a^
pH	6.26 ± 0.21 ^a^	4.96 ± 0.12 ^c^	5.24 ± 0.11 ^b^
LA, mg/g	22.19 ± 1.19 ^b^	41.09 ± 1.34 ^a^	40.99 ± 1.31 ^a^
AA, mg/g	0.74 ± 0.04 ^c^	5.85 ± 0.12 ^b^	8.75 ± 0.19 ^a^
PA, mg/g	0.22 ± 0.01 ^c^	0.61 ± 0.03 ^b^	0.70 ± 0.03 ^a^
BA, mg/g	2.81 ± 0.16 ^a^	1.15 ± 0.13 ^c^	2.11 ± 0.15 ^b^

^1^ CK group: Feed 100% basal diet; YR1 group: Feed 50% basal diet + 50% *Bacillus amyloliquefaciens* YR3.2 fermented feed; YR2 group: Feeding 50% basal diet + 50% *Bacillus licheniformis* YR3.1 fermented feed. ^a–c^ Different lowercase letters indicate significant differences (*p* < 0.05), and the same letters or no letters indicate no significant differences (*p* > 0.05). DM, dry matter; CP, crude protein; CF, crude fiber; NDF, neutral detergent fiber; ADF, acid detergent fiber; NH_3_-N, ammonia nitrogen; SS, soluble sugar; ST, starch; LA, lactic acid; AA, acetic acid; PA, propionic acid; BA, butyric acid.

**Table 3:** Effects of Fermented Feed on the Growth Performance and Carcass Traits of Fattening Pigs.

Item	CK	Treatment		*p*-Value
		YR1	YR2	
Initial weight, kg	68.67 ± 0.94	68.87 ± 0.97	68.49 ± 0.58	0.825
Final weight, kg	96.54 ± 0.96 ^c^	109.27 ± 0.95 ^a^	99.89 ± 1.65 ^b^	<0.001
Average daily feed intake (ADFI), kg	2.62 ± 0.08	2.59 ± 0.05	2.60 ± 0.07	0.821
Average daily gain (ADG), kg	0.56 ± 0.04 ^c^	0.81 ± 0.02 ^a^	0.63 ± 0.03 ^b^	<0.001
Feed-to-gain ratio (F/G)	4.71 ± 0.32 ^a^	3.21 ± 0.12 ^c^	4.20 ± 0.24 ^b^	<0.001
Carcass weight (CW), kg	66.02 ± 4.67 ^b^	76.84 ± 2.00 ^a^	71.31 ± 5.70 ^b^	0.008
Dressing percentage, %	68.41 ± 5.39	70.33 ± 2.07	69.97 ± 4.14	0.636
Average backfat thickness, mm	23.10 ± 0.31 ^a^	21.28 ± 1.96 ^ab^	19.79 ± 0.24 ^b^	0.013

^a–c^ Different lowercase letters indicate significant differences (*p* < 0.05), and the same letters or no letters indicate no significant differences (*p* > 0.05). CK group: Feed 100% basal diet; YR1 group: Feed 50% basal diet + 50% *Bacillus amyloliquefaciens* YR3.2 fermented feed; YR2 group: Feeding 50% basal diet + 50% *Bacillus licheniformis* YR3.1 fermented feed.

**Table 4:** Effects of Fermented Feed on the Meat Quality of Fattening Pigs.

Item		CK	Treatment		*p*-Value
			YR1	YR2	
	L*	41.79 ± 0.29	41.32 ± 0.44	41.51 ± 0.93	0.572
Meat color	a*	4.11 ± 0.16 ^b^	6.66 ± 0.32 ^a^	6.39 ± 0.08 ^a^	<0.001
	b*	5.88 ± 0.23	6.12 ± 0.07	6.12 ± 0.12	0.113
pH _45min_		6.37 ± 0.14	6.33 ± 0.16	6.17 ± 0.08	0.208
pH _24h_		5.60 ± 0.10 ^a^	5.36 ± 0.14 ^b^	5.67 ± 0.09 ^a^	0.021
Shear force, N		42.76 ± 1.50 ^a^	34.61 ± 2.35 ^b^	37.62 ± 2.05 ^b^	0.020
Cooked meat rate, %		68.07 ± 1.17	68.08 ± 0.82	68.58 ± 1.61	0.459
Water loss rate, %		5.43 ± 0.41 ^a^	4.55 ± 0.53 ^b^	5.24 ± 0.29 ^a^	0.041

^a–b^ Different lowercase letters indicate significant differences (*p* < 0.05), and the same letters or no letters indicate no significant differences (*p* > 0.05). CK group: Feed 100% basal diet; YR1 group: Feed 50% basal diet + 50% *Bacillus amyloliquefaciens* YR3.2 fermented feed; YR2 group: Feeding 50% basal diet + 50% *Bacillus licheniformis* YR3.1 fermented feed. L*, lightness; a*, redness; b*, yellowness.

**Table 5:** Effect of Fermented Feed on Serum Biochemical Indices of Fattening Pigs.

Item	CK	Treatment ^1^		*p*-Value
		YR1	YR2	
Glu, mmol/L	5.67 ± 1.46	6.00 ± 0.39	6.03 ± 0.58	0.836
TC, mmol/L	2.52 ± 0.23	2.44 ± 0.09	2.65 ± 0.19	0.293
TG, mmol/L	0.39 ± 0.09	0.40 ± 0.08	0.38 ± 0.18	0.975
TP, g/L	72.93 ± 2.60	77.43 ± 0.84	75.80 ± 4.16	0.136

^1^ CK group: Feed 100% basal diet; YR1 group: Feed 50% basal diet + 50% *Bacillus amyloliquefaciens* YR3.2 fermented feed; YR2 group: Feeding 50% basal diet + 50% *Bacillus licheniformis* YR3.1 fermented feed. Glu, glucose; TC, total cholesterol; TG, triglyceride; TP, total protein.

**Table 6:** Effects of Fermented Feed on the Serum Antioxidant Function of Fattening Pigs.

Item	CK	Treatment		*p*-Value
		YR1	YR2	
SOD, U/mL	343.34 ± 10.13 ^b^	383.30 ± 2.41 ^a^	385.04 ± 28.46 ^a^	0.020
MDA, nmol/mL	3.29 ± 0.38	3.43 ± 0.14	3.05 ± 0.29	0.211
CAT, U/mL	4.61 ± 0.31	5.84 ± 1.03	5.23 ± 1.11	0.112
T-AOC, mM	0.35 ± 0.04	0.36 ± 0.04	0.36 ± 0.03	0.899

^a–b^ Different lowercase letters indicate significant differences (*p* < 0.05), and the same letters or no letters indicate no significant differences (*p* > 0.05). CK group: Feed 100% basal diet; YR1 group: Feed 50% basal diet + 50% *Bacillus amyloliquefaciens* YR3.2 fermented feed; YR2 group: Feeding 50% basal diet + 50% *Bacillus licheniformis* YR3.1 fermented feed. SOD, superoxide dismutase; MDA, malondialdehyde; CAT, catalase; T-AOC, total antioxidant capacity.

**Table 7:** Effects of Fermented Feed on Serum Immune Parameters of Fattening Pigs.

Item	CK	Treatment		*p*-Value
		YR1	YR2	
IgA, μg/mL	8.24 ± 0.23	8.40 ± 0.26	8.99 ± 0.84	0.257
IgG, mg/mL	1.67 ± 0.42 ^b^	1.81 ± 0.03 ^b^	2.46 ± 0.36 ^a^	0.024
IgM, mg/mL	0.83 ± 0.17	0.99 ± 0.16	1.07 ± 0.11	0.220
IL-2, pg/mL	41.34 ± 2.65 ^b^	44.45 ± 2.07 ^b^	52.79 ± 6.21 ^a^	0.014
IL-6, pg/mL	104.79 ± 9.17	99.81 ± 13.73	103.02 ± 7.71	0.845
IL-10, pg/mL	23.04 ± 1.82 ^b^	28.00 ± 2.33 ^a^	30.19 ± 2.19 ^a^	0.016
TNF-α, pg/mL	11.42 ± 2.06 ^a^	9.53 ± 0.82 ^b^	11.14 ± 0.87 ^a^	0.026

^a–b^ Different lowercase letters indicate significant differences (*p* < 0.05), and the same letters or no letters indicate no significant differences (*p* > 0.05). CK group: Feed 100% basal diet; YR1 group: Feed 50% basal diet + 50% *Bacillus amyloliquefaciens* YR3.2 fermented feed; YR2 group: Feeding 50% basal diet + 50% *Bacillus licheniformis* YR3.1 fermented feed. TNF-α, tumor necrosis factor.

**Table 8:** Reagents and consumables used for feed fermentation and estimated fermentation cost per pig.

Reagents and Consumables	Amount Used	Total Cost (CNY)
Tryptone (500 g)	15 bottles	4140
Yeast extract (500 g)	8 bottles	1344
1.5 mL centrifuge tubes (500 pieces)	2 packages	23
NaCl (500 g)	15 bottles	225.6
Agar powder (250 g)	1 bottle	36.9
NaOH (0.1 mol/L)	1 bottle	57.9
HCl (0.1 mol/L)	1 bottle	65
Fermentation bags (50 kg)	340 bags	553.9
Total		6446.3

**Table 9:** Estimated income before deduction of fermentation cost (CNY/head).

Group	Mean Final Body Weight (kg)	Average Feed Intake over ^1^ 50 d (kg/Head)	Net Income (CNY/Head)	Increased Income Compared with CK (CNY/Head)
CK	96.54	131.00	993.32	---
YR1	109.27	129.50	1188.51	+195.19
YR2	99.89	130.00	1046.76	+53.44

^1^ ADFI and economic calculation were calculated according to dry matter or equal dry matter intake.

## Data Availability

The datasets generated during the current study are available from the corresponding author on reasonable request.

## Abbreviations

The following abbreviations are used in this manuscript:

AA	Acetic acid
ADF	Acid detergent fiber
ADMI	Average daily feed intake
ADG	Average daily gain
BA	Butyric acid
CAT	Catalase
CF	Crude fiber
CP	Crude protein
CW	Carcass weight
DE	Digestible Energy
DM	Dry matter
FBW	Final body weight
FCR	Feed conversion ratio
Glu	Glucose
Ig	Immunoglobulin
IL	Interleukin
IBW	Initial body weight
LA	Lactic acid
MDA	Malondialdehyde
NDF	Neutral detergent fiber
NH_3_-N	Ammonia nitrogen
PA	Propionic acid
SOD	Superoxide dismutase
SS	Soluble sugar
ST	Starch
T-AOC	Total antioxidant capacity
TC	Total cholesterol
TG	Triglyceride
TNF-α	Tumor necrosis factor
TP	Total protein
